# Interstitial inflammation and pulmonary fibrosis in COVID-19: The potential role of cytostatic therapy for severe lung injury

**DOI:** 10.1016/j.rmcr.2022.101676

**Published:** 2022-05-27

**Authors:** Elena V. Filimonova, Lyubov A. Davydova, Mariana A. Lysenko, Sergey V. Tsarenko

**Affiliations:** a State Budgetary Healthcare Institution “Moscow City Clinical Hospital № 52 of Moscow Healthcare Department” (MCCH52), 3 Pekhotnaya Street, Moscow, 123182, Russia; b Lomonosov Moscow State University, Faculty of Medicine, 27/10 Lomonosovsky Prospekt, Moscow, 119991, Russia; c Chief Medical Officer of City Clinical Hospital No. 52 of Moscow Healthcare Department, 3 Pekhotnaya Street, Moscow, 123182, Russia; d Department of Internal Medicine, Faculty of Additional Professional Education at Federal State Autonomous Educational Institution of Higher Education Pirogov Russian National Research Medical University, 1 Ostrovitianov Street, 117997, Moscow, Russia; e Deputy Chief Medical Officer in Anesthesiology and Intensive Care at City Clinical Hospital No. 52 of Moscow Healthcare Department, 3 Pekhotnaya Street, 123182, Moscow, Russia; f Department of Anesthesiology and Reanimatology at Lomonosov Moscow State University, Faculty of Medicine, 27/10 Lomonosovsky Prospekt, Moscow, 119991, Russia

**Keywords:** COVID-19, Pulmonary fibrosis, Respiratory failure, Cytokine storm, Cyclophosphamide, Extracorporeal membrane oxygenation

## Abstract

The progression of secondary pulmonary damage in SARS-COV-2 infection, associated with interstitial damage, inflammation and alveolar consolidation and eventually resulted in the development of pulmonary fibrosis (PF), remains one of the key clinical dilemmas for the treatment of patients in intensive care units (ICU). Currently, there is no standardized algorithm for PF prevention and timely management, although few studies have discussed the use of antifibrotic agents in COVID-19 patients.

One of the treatment options for patients with interstitial PF, when irresponsive to the given corticosteroid therapy, is the administration of cytostatic agents, in particular, cyclophosphamide. Cyclophosphamide is one of the well-studied drugs in the cytostatic group, which effectiveness in inhibiting systemic inflammation suggests its ability to reduce the progression of the secondary lung damage, interstitial abnormalities and PF caused by the so-called “cytokine storm".

The presented case reports provide data on the use of cyclophosphamide (Сy) in the management of severe respiratory failure in COVID-19 patients stationed in ICU. We describe three clinical cases characterized by different types of respiratory support, including extracorporeal membrane oxygenation.

## Highlights

• Cyclophosphamide can be considered relatively safe and reasonably effective in severe lung damage in COVID-19.
• Cyclophosphamide may reduce interstitial inflammation, thus preventing the development of pulmonary fibrosis.
• No severe side effects were observed during low-dose cyclophosphamide therapy in our study

## 1 Introduction

Declared by the World Health Organization (WHO) on March 11, 2020, the COVID-19 pandemic continues to pose a significant challenge for public health at present: the questions of pathogenesis, diagnosis, and treatment of the novel coronavirus infection are widely discussed in Russian and international medical literature[1].

Disease severity and hospitalization rate of COVID-19 are higher in elderly people and patients with chronic comorbidities, notably, arterial hypertension, diabetes mellitus, coronary heart disease, and chronic obstructive pulmonary disease (COPD). So far, the globally accumulated facts are as follows: male patients demonstrate a higher predisposition to severe COVID-19 [1]; the mortality rate is less than 5% in patients aged <80 without comorbidities [2]; 50% of those infected have asymptomatic course of the disease; if presenting clinical symptoms, 80% of patients have only mild form of acute respiratory infection.

The most frequent COVID-19 manifestation is a bilateral pneumonia, which accounts for 3–4% of the cases, with subsequent progression to severe acute respiratory distress syndrome (ARDS) [3]. According to Grasseli et al., 40–96% of ARDS patients died in intensive care units (ICU) in Lombardy, Italy, which makes ARDS one of the major risk factors associated with mortality among COVID-19 patients in ICUs [1]. In the first 2 months of the pandemic, ICU and in-hospital mortality rates were 48.8% and 53.4%, respectively, due to the demand for prolonged mechanical ventilation in those patients. In a series of studies conducted in China, 28-day mortality rate of ICU patients was 39%, with up to 97% in the ICU patients on mechanical ventilation [1]. The main cause of death in ICU patients was sepsis. Among the most frequent autopsy findings, there were also suppurative lung disease, diffuse alveolar damage, pulmonary embolism, cardiopulmonary, and multiple organ system failure [2].

The international clinical guidelines and approved treatment strategies for ICU patients comprise symptomatic therapy with concomitant conditions in consideration, administration of antiviral and immunomodulatory drugs as well as pathogen-specific therapy [3]. However, secondary pulmonary damage, associated with interstitial inflammation and alveolar consolidation, commonly develops in case of severe course of the disease and eventually results in, that is usually referred to, as pulmonary fibrosis (PF) [5]. Currently, there is no standardized algorithm for PF prevention and its timely management, which makes it one of the key clinical dilemmas for the treatment of ICU patients.

Despite the novelty of the problem, few studies have discussed the use of antifibrotic agents in COVID-19 patients. ERS guidelines recommended nintedanib and pirfenidone for the treatment of idiopathic pulmonary fibrosis (IPF) in 2015 [12]. Earlier these drugs showed their effectiveness in preventing the decline in forced vital capacity (FVC), decreasing IPF exacerbations and hospital admissions in those patients [4].

A similar clinical and radiological picture can be observed in patients with organizing pneumonia (OP).

Firstly, OP demonstrates the same CT pattern as in COVID-19-associated severe lung damage, so-called ground-glass opacities, consolidation, reticulation, and parenchymal distortion of 70–90% of lung volume. OP comprises granulation tissue and proliferation of fibroblasts within the lung parenchyma. Viral infections are amongst the most common etiologies of secondary OP. Secondly, OP often has dramatic reversibility when managed with corticosteroids, therefore presents a beneficial treatment option in severe forms of COVID-19 pneumonia and late phase post-pneumonic complications [6,7]. Generally, corticosteroids remain the mainstay for interstitial lung disease therapy, both in mono- and combination (with cytostatic agents) therapies [8].

The administration of cytostatic agents, in particular, cyclophosphamide (Cy), mycophenolate [[8], [9], [10]], and antitumor drugs [12,13] may present an additional therapy option for patients with PF and OP, when irresponsive to the given therapy. Cyclophosphamide is one of the most studied drugs in the cytostatic group, which effectiveness in inhibiting systemic inflammation suggests its ability to reduce the progression of the secondary lung damage, interstitial abnormalities and PF caused by the so-called “cytokine storm” [10,11,14].

However, the major concern regarding the indication of cytostatic agents is the wide range of its adverse effects. Clinicians need to consider key aspects of Cy pharmacodynamics and toxicity to adequately assess the risk-benefit ratio. Prospective studies in this field may admit Cy with respect to its side effects as an inexpensive option to treat severe COVID-19-associated pulmonary fibrosis.

## 2 Case reports

Here we present several clinical cases of the use of Cy in the management of severe respiratory failure in COVID-19 patients stationed in ICU. The off-label use of low-dose Cy was approved by the local ethics committee of the clinical hospital.

### 2.1 Case 1

A 46-year-old male (BMI 30.8 kg/m^2^) presented on non-invasive ventilation (NIV) with severe respiratory failure and dyspnea, was admitted to ICU on Day 30 from symptoms onset. Previously, he was treated with antibiotics (ceftriaxone, levofloxacin, vancomycin, and meropenum), antivirals (oseltamivir, favipiravir), olokizumab 64 mg, and prednisolone for 7 days in cumulative dose of 1640 mg. On day 30, the patient had a positive PCR-test with oropharyngeal swab for SARS-COV-2 and progressive respiratory failure. The characteristics of NIV were as follows: FiO_2_ - 90%, SpO_2_ - 90–93%, prone position, breath rate - 20–24/min. Due to exercise intolerance, the patient totally avoided physical activity. Laboratory findings showed elevated LDH 785 U/L and C reactive protein 56.52 mg/L, lymphopenia 0.5*10^9^/L, D-dimer 1692 ng/mL. Admission CT findings showed the volume ratio of 90% ground-glass opacity.

ICU therapy commenced on enoxaparin (antiXa guided with target 0.4–0.6), dexamethasone 24 mg/daily . Despite the late inflammatory phase (Day 30), it was decided to infuse a single dose of tocilizumab 400 mg combined with olokizumab 128 mg. Therapeutic plasmapheresis was performed the following day. For 10 days, the patient stayed in the prone position on NIV with FiO_2_ 90% or high-flow oxygen (HFO) with FiO_2_ 95% to maintain SpO_2_ 90–93%. CT scan performed on Day 45 showed fibrous stripes and reticular patterns. Owing to no clinical improvement, we started intravenous cytostatic antifibrotic therapy with Cy 100 mg daily. A ‘stop-dose’ of 2000 mg was agreed, with earlier cancellation depending on clinical efficacy or signs of systemic toxicity.

**Fig. 1:**
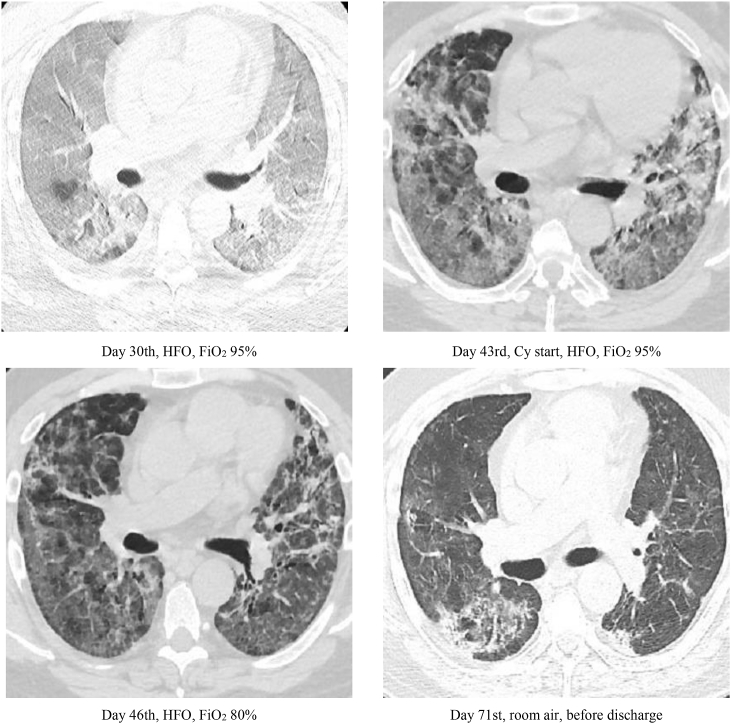
Lung CT scans of Patient 1 (days of the disease).

On Day 5 of Cy therapy, the patient's oxygenation level improved dramatically, his physical activity increased as well. On Day 10 in ICU, HFO was replaced with low-flow oxygen. The patient was discharged from ICU to the hospital general ward. Cy infusions were abandoned on Day 17 with the total dose of 1700 mg, followed by the patient's discharge in three weeks without oxygen support. No severe side effects or organ toxicity during the Cy treatment were observed. Minimal leucocyte level was 5.4*10^9^/L, platelets - 135*10^9^/L, controlled daily. Suggesting the leukocytosis level up to 17*10^9^/L and positive sputum culture for *Klebsiella pneumoniae* and Sternotrophomonas maltophilia, antibiotics, namely, co-trimoxasol 1880 mg and gentamycin 480mg were administered daily for 2 weeks (Fig. 1).

### 2.2 Case 2

**Fig. 2:**
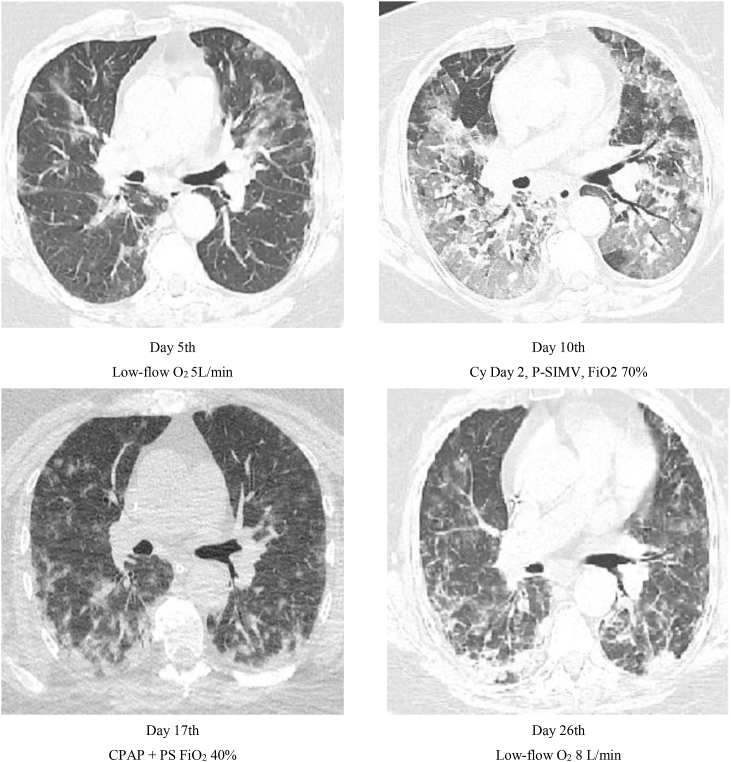
Lung CT scans of Patient 2.

A 62-year-old female (BMI 33.8 kg/m^2^) was admitted to ICU on Day 8. Onset symptoms were high temperature, weakness, dry cough, and diarrhea. She was tested positive with PCR-oropharyngeal swab for SARS-COV-2. She was hospitalized with mild respiratory failure on Day 5 from the onset of symptoms. On admission, she was in need of 4 L/min low-flow oxygen to achieve SpO_2_ 95–98% without dyspnea. Ground-glass opacities and ‘crazy paving’ accounted for 30% of the lung tissue according to Day 5 CT scan. Admission lab findings revealed CRP 62.6 mg/L, LDH 305.6 U/L, lymphopenia 0.5х10*9/L. The patient was administered amoxicillin/sulbactam, levofloxacin, enoxaparin, dexamethasone 16 mg/daily. On Day 5 the patient commenced on tocilizumab 400 mg and levilimab 486 mg. On Day 7, the patient received 320 ml of convalescent plasma. On Day 9, owing to the progressive respiratory failure, the patient was indicated HFO with FiO2 80%, Flow 50l/min combined with Cy therapy of 200 mg/daily. Invasive ventilation was started on Day 10, with percutaneous tracheostomy performed. CT scan revealed the volume ratio of ground-glass opacity, exceeding 80%, as well as ‘crazy paving’ with the reticular pattern. It was decided to maintain daily Cy therapy of 200 mg, the dose of which was decreased to 100 mg/daily on Day 12. Antimicrobial treatment with polymyxin 300 mg/daily was given on Day 13. The total amount of Cy received was 1200 mg. Dexamethasone was used for 14 days at dose of 16 mg/daily. The patient's respiratory condition improved dramatically, the weaning from ventilator support was conducted on day 10 in ICU. Starting from Day 20, the patient breathed successfully with the flow of 5 L/min. Decannulation was followed by the patient's transfer to the general ward. On day 60, the patient was discharged with the significantly improved level of physical activity and no need for oxygen support (Fig. 2).

We did not observe organ toxicity during the Cy treatment. Minimal leucocyte level was 3.1 *10^9^/L, platelets - 76*10^9^/L, daily controlled

### 2.3 Case 3

A 24-year-old male (BMI 21.6 kg/m^2^) on mechanical ventilation with severe respiratory failure due to lab-confirmed COVID-19-associated bilateral pneumonia, with no comorbidities, was transferred to the ICU from a different hospital.

The onset of the disease was characterized by high temperature of 38.5 and cough, no specific treatment was administered. On day 4 of the disease, the patient's respiratory symptoms worsened, which caused his hospitalization. CT scan showed a specific pattern of massive bilateral ground-glass opacities (CT-2). Due to the high procalcitonin level (4.5), immunosuppressive therapy with tocilizumab was abandoned, whereas antimicrobial therapy (cefepime, levofloxacin) was indicated. However, the patient's condition continued to deteriorate, and invasive mechanical ventilation was initiated on day 8 of the disease. CT scan showed the progression of pneumonia to CT-4 (increased number of ground-glass opacity zones combined with consolidation). Due to low index p/F 82 and non-protective ventilation, the patient was transferred to the Moscow City Clinical 52 ECMO center. Laboratory tests showed high CRP 66.51 mg/L, WBC 6.5, LDH 722.6 U/L, lymphocytes 0.3х10*9/L, PCT 0.54 ng/ml. The clinical features coupled with laboratory and radiological findings revealed cytokine storm progression. According to the previous findings, the patient received a single dose of tocilizumab 400 mg (day 9 of the disease). The lung CT of day 9 showed bilateral ground-glass opacities combined with consolidation and ‘crazy-paving’ pattern. Owing to the ongoing respiratory deterioration and poor radiological findings, Cy therapy of 100 mg/daily was started on admission to ECMO ICU.

**Fig. 3:**
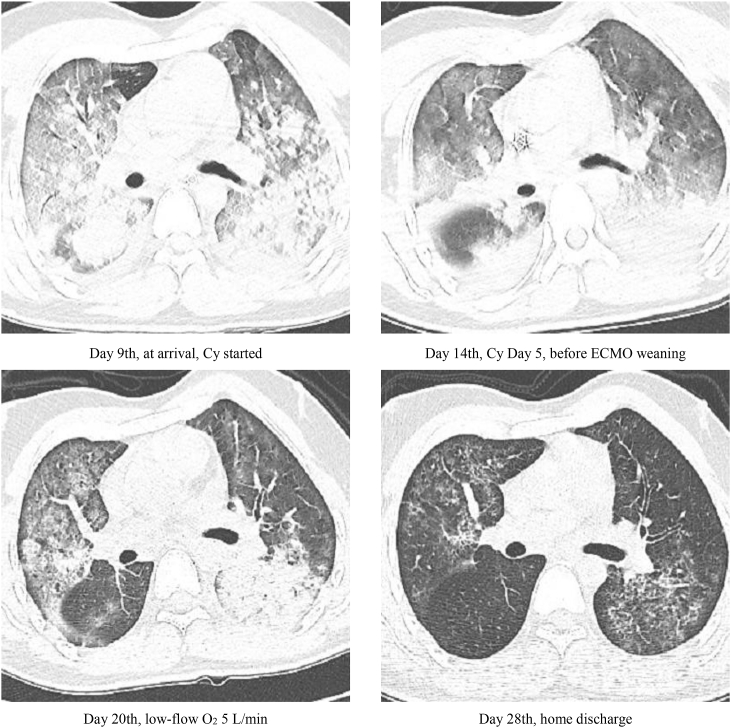
Lung CT scans of Patient 3.

VV-ECMO together with shifting to lung-protective ventilation was initiated on day 10. Three days later tracheostomy was applied and myoplegia was prevented. The patient was emerged and demonstrated an adequate lung contribution (increased Vt and compliance). In 12 hours, ECMO parameters were reduced to the minimum, which resulted in his discharge from ECMO. On day 18 from the disease onset, the patient was decannulated. He was provided with 5 L/min oxygenation for the subsequent 5 days. On day 28 from the start of the disease, the patient was discharged with no oxygen dependence (Fig. 3).

Starting from day 9 of the disease onset for the duration of 13 days, the patient was administered cumulatively 1300 mg of Cy, with no signs of systemic toxicity observed. Minimal number of WBC during the hospitalization was 3.4* 10^9^/L and number of platelets was 148*10^9^/L.

## 3 Discussion

According to the accumulated data, we suggest using Cy as a treatment option for patients not responding to corticosteroids or IL-6 inhibitors. Cy should be used only in case of severe forms of respiratory distress considering all contraindications, such as cytopenia, sepsis, and pregnancy. We successfully used Cy in patients receiving different respiratory support, though strongly advocating for acknowledging the risk of secondary bacterial ventilator-associated pneumonia in ventilated patients. In the presented above cases, all mechanically ventilated patients had received antibiotics prior to the Cy administration and improved rapidly without ventilator-associated pneumonia progression. We do not recommend the administration of prophylactic antibiotics in patients on high flow oxygenation or non-invasive ventilation and administering ABT only in presence of the infection criteria (e.g., fever, positive blood culture, increased sputum production, leukocytosis, new radiological findings). Thrombocytopenia and leucopenia require a thorough monitoring throughout the whole period of Cy administration, and we recommend to look after blood cell count in approximately 7-10 days after the course of Cy therapy is finished. Additionally, we suggest lower doses of Cy in elderly patients in comparison to younger ones on account of existing age-related myelosuppression and withdrawal of Cy treatment in signs of myelosuppression or bacterial/fungal infection progression.

## 4 Conclusion

Therefore, a low-dose Cy therapy may be considered relatively safe and reasonably effective in COVID-19 patients with severe lung injury and deteriorating respiratory conditions. It may help to reduce interstitial inflammation and alveolar consolidation, which eventually cause pulmonary fibrosis. Further investigation and research on animals and humans are necessary.

## Declaration of competing interest

We declare no competing interests.
